# A Study to Assess the Role of Date Syrup, Pineapple Juice, and Hematinic Syrup as Magnetic Resonance Oral Contrast Agents in Improving the Image Quality of Magnetic Resonance Cholangiopancreatography

**DOI:** 10.7759/cureus.57769

**Published:** 2024-04-07

**Authors:** Karpagam R K, Pujitha Duvooru Sukumar, Pranathi Ravula, Karthik Krishna Ramakrishnan, Shree Haritha P, Paarthipan Natarajan

**Affiliations:** 1 Department of Radiology, Saveetha Medical College and Hospital, Saveetha Institute of Medical and Technical Sciences (SIMATS) Saveetha University, Chennai, IND

**Keywords:** hematinic syrup, date syrup, pineapple juice, bile duct, mr cholangiopancreatography

## Abstract

Introduction: Magnetic resonance cholangiopancreatography (MRCP) is an imaging technique that has advanced over the past few years. It still plays a crucial role in the study of numerous pancreaticobiliary diseases. This study aimed to evaluate the effects of hematinic syrup, date syrup, and pineapple juice on MRCP image quality.

Methodology: This study involved a total of 90 participants, distributed evenly among three groups, with each group comprising 30 patients. Negative oral contrast solutions containing paramagnetic substances like Mn+2 and Fe+3, such as pineapple juice, date syrup, and hematinic syrup were imaged by 1.5 Tesla (T) magnetic resonance imaging (MRI) with T2-weighted (T2W) and MRCP sequences. The signal-to-noise ratio (SNR) and contrast-to-noise ratio (CNR) were computed. Ninety patients underwent MRCP 20-30 min after ingestion of 100 mL of date syrup, 30 ml of hematinic syrup diluted to 200 ml of water, and 200 mL of pineapple juice. MRCP images were taken to visualize various pancreaticobiliary structures (bile duct, stomach, and duodenum).

Results: The in vitro evaluation of the solutions showed that date syrup and hematinic syrup were hypointense in T2W sequences. The images obtained showed no significant difference in the CNR between the three solutions. However, the SNR was significantly higher for pineapple juice compared to date syrup and hematinic syrup in T2W and MRCP sequences. Images acquired post-administration of the oral contrast agents significantly improved the gastrointestinal tract signal suppression and increased visibility of the pancreaticobiliary structures (bile duct, stomach, and duodenum). No adverse events were observed among the participants.

Conclusion: Pineapple juice was the best contrast agent. However, date syrup and hematinic syrup can also be used to improve the imaging quality.

## Introduction

Magnetic resonance cholangiopancreatography (MRCP) is a noninvasively used method for visualizing the pancreatic ductal system and the intra- and extrahepatic biliary tree [1]. When MRCP was first introduced to the clinical setting by Wallner et al. [2] in 1991, it was viewed as a secondary-level examination for the diagnosis of obstructive jaundice [1]. During the last two decades, this MRCP has greatly expanded its diagnostic role in diagnosing numerous pathologic conditions [2-4].

MRCP makes use of heavily T2-weighted (T2W) sequences to make most of the characteristic contrast nature of the bile juice [5]. The potential overlap of the static fluids in the pancreatobiliary system and the gastrointestinal tract (the stomach, duodenum, and proximal jejunum) is a major drawback of MRCP. This overlap can conceal the distal part of the common bile duct or mimic an illness [6]. To work around this constraint, pharmaceutical companies have produced several oral negative contrast agents that contain paramagnetic substances that shorten the T2 relaxation time. This reduces the signal hyperintensity of gastroenteric fluids in standard MRCP sequences. These agents have been used regularly in radiological practice in the past [7, 8]. These negative oral contrast agents, however, might not be palatable, dilute excessively much in the gut, or cost too much [7].

The oral contrast agent should not induce peristalsis or dilute in GI fluid; instead, it should be nontoxic, reasonably priced, palatable, and must be distributed uniformly throughout the GI tract. It should be diluted during transit or stimulate peristalsis [9]. The search for more affordable, tolerable, and widely used natural contrast agents in the commercial sector is still ongoing [10]. Naturally available fruit juices, among which the most well-known are pineapple juice and blueberry juice, have drawn attention in more recent times because of their lower cost and higher palatability, as well as their similar ability to suppress the high T2 signal from GI tract liquids caused by high manganese and iron content [8]. Iron-rich sources such as date syrup [11] and ferric ammonium citrate [12]-based contrast agents have been used as safe and efficient contrast agents for the GI tract.

Hence, this study aimed to assess the role of date syrup, pineapple juice, and hematinic syrup as MR oral agents in improving the image quality of MRCP with the oral solutions acting as negative contrast agents. Also, in vitro and qualitative analysis of the quality of the MRCP images for various contrast agents was done.

## Materials and methods

This study was a prospective study conducted between January 2019 and December 2020 on 90 healthy volunteers at Saveetha Medical College Hospital, Thandalam, Chennai. The institutional review board of Saveetha Medical College Hospital approved this study. The study was conducted in accordance with the Declaration of Helsinki (as revised in 2013). All apparently healthy volunteers with no abnormality in the biliary system attending the Department of Radiology at Saveetha Medical College Hospitals were included in the study. Volunteers with incidental findings, studies with poor anatomical delineation, and claustrophobic tendencies were excluded. Individuals with contraindications such as cardiac pacemaker, cochlear implants, metal implants, dentures, and pregnancy were disqualified from participation. Patients were randomly allocated into three distinct groups, with each group receiving a unique oral contrast agent.

The initial group of patients, numbering 30, was administered with pineapple juice. Each 200 ml serving contained concentrated pineapple juice and natural fruit sugars (8.5%), recognized for their paramagnetic properties due to manganese presence. Additionally, the nutritional content per serving included 11 g of carbohydrates, 10.6 g of sugar, 0.2 g of protein, 22 mg of sodium (paramagnetic), and 75 mg of potassium (paramagnetic).

The second set of patients, also consisting of 30 individuals, was given date (*Phoenix dactylifera*) syrup. For every 100 ml of syrup, the composition included 1.54 mg of iron (ferromagnetic), 6.24 mg of calcium (paramagnetic), 121 mg of potassium (paramagnetic), 33.2 mg of sodium (paramagnetic), 5.63 g of dietary fiber, and 80 g of carbohydrates.

The third group, comprising 30 participants, was administered with hematinic syrup characterized by an orange-flavored formula used as a negative oral contrast agent. This syrup is a blend of vitamins and iron supplements. Each tablespoon (15 ml) of hematinic syrup contains the following constituents: 7.5 mg of cyanocobalamin (ferromagnetic), 0.5 mg of vitamin B9 (folic acid), 160 mg of ferric ammonium citrate (paramagnetic), and 0.87 ml of alcohol (95% IP). Either 100 mL of date syrup or 30 mL of hematinic syrup diluted to 200 mL with water, or 200 mL of pineapple juice, was consumed by the patients. After the patient ingested it, we had to wait half an hour for it to coat the duodenum and stomach. To avoid repetitive swallowing, patients were asked to consume a bolus of the oral contrast agent, swallow it in one gulp, and then open their mouths afterward.

MR imaging (MRI) was conducted using a Philips Multiva 1.5 Tesla (T) MRI machine, equipped with a bellows setup around the chest for imaging the chest and abdomen. Prior to the MRI examination, a clinician recorded the patient's medical history and assessed their swallowing capability in a supine position. Written informed consent was secured from each patient before they were allowed into the scanner room. Participants were instructed to fast for 4-6 hours preceding the examination. They were also required to remove all metallic objects and implants before the scan. Patients received a thorough explanation of the procedure in advance.

The patient was positioned in a supine position with head-first orientation. Then, the patient was positioned over the spine coil, and the body coil was placed over the upper abdomen. The body coil was securely tightened using straps to prevent respiratory artifacts. The laser beam localizer was centered over the xiphoid process of the sternum.

Instructions for holding breath were provided to the patients, and surface coils were utilized to capture the images. Two imaging techniques were employed: one requiring a breath-hold with a single-shot method and the other, a non-breath-hold technique using respiratory triggering. Image acquisition was performed in both two-dimensional (2D) and three-dimensional (3D) formats. For sequences of longer duration, respiratory gating was implemented to mitigate respiratory ghosting effects, ensuring that each image slice was captured at a consistent respiratory phase.

**Figure 1:**
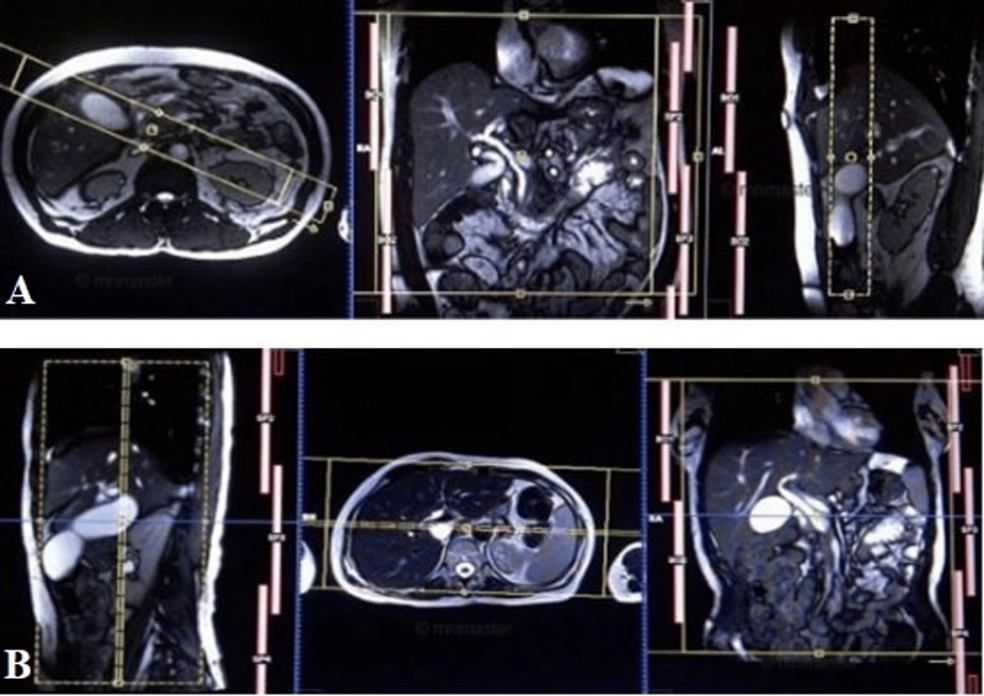
Optimizing T2-weighted MRCP imaging: Axial and coronal planning strategies (A) To plan the T2-weighted axial section of the gallbladder (GB) with a yellow dashed box in MRCP, ensure the imaging slice is positioned parallel to the GB's long axis for comprehensive visualization. Adjust slice thickness and orientation to optimize clarity of the GB and surrounding structures, including the common bile duct and the liver. (B) To plan the T2-weighted coronal section of the gallbladder (GB) with a yellow dashed box in MRCP, ensure the imaging slice is positioned perpendicular to the GB's long axis for comprehensive visualization. Adjust slice thickness and orientation to optimize clarity of the GB and surrounding structures, including the common bile duct and the liver.

Figure 1 illustrates the crucial planning strategies for optimizing T2W MRCP imaging. Through both axial and coronal perspectives, this figure delineates the strategic orientations necessary to capture detailed images of the biliary and pancreatic systems. Such meticulous planning ensures a comprehensive visualization and accurate diagnosis, thereby enhancing the clinical utility of MRCP in detecting various hepatobiliary and pancreatic pathologies.

**Figure 2:**
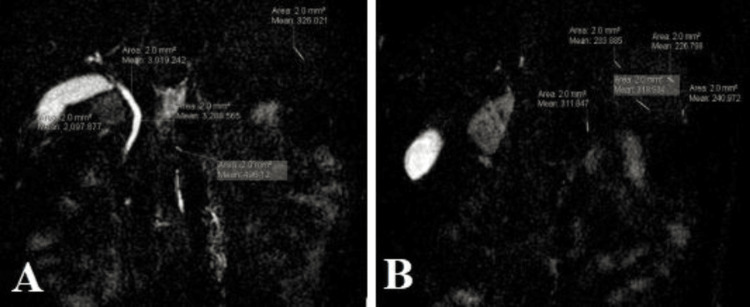
Measurement of the CNR and the SNR (A) The contrast-to-noise ratio (CNR) at the gallbladder (GB) level in the MRCP indicates how well the scan distinguishes between structures in the image and the background noise. It reflects the MRCP's capacity to differentiate tissues like the GB, bile ducts, and nearby organs amid noise interference. A higher CNR suggests enhanced contrast and better ability to detect abnormalities. (B)The signal-to-noise ratio (SNR) at the level of the gallbladder (GB) in the MRCP refers to the ratio of the signal intensity from the desired anatomical structures, such as the bile ducts and the GB itself, to the background noise present in the image. A higher SNR indicates better image quality and improved ability to discern fine details and abnormalities within the GB and the surrounding structures.

Two radiologists independently examined the MR images. Each radiologist evaluated an MR image in a random order after being blinded to the assessments of the others. To prevent interactions, each of the three MR parameters was assessed in a different session. Signal-to-noise ratio (SNR): The technique to obtain SNR values is done by placing the region of interest (ROI) on the stomach (Figure 2a). SNR standard = average signal of the organ/standard deviation of the noise, wherein signal is the signal intensity measured in the ROI ellipse, and noise (SD) is the basic noise defined as the standard deviation of the measurements made, by placing a circle 2 cm (ROI) anterior to the abdominal wall (air). SD = √∑(x − μ)2/n, where SD = population standard deviation, n = size of the population, x = each value from the population, and μ = the population mean. The technique to obtain contrast-to-noise-ratio (CNR) values is done by placing the ROI on the stomach, biliary tree, and duodenum (Figure 2b). The CNR is just the ratio of the estimated contrast and noise. CNR = ROI organ-ROI background/standard deviation of noise (Figure 2, Table 1).

**Table 1:** Parameters of the MRCP MRI TR: Time to repetition; TE: time to echo; TSE: turbo spin-echo; SNR: signal-to-noise ratio; NSA: number of signal averages; MRCP: magnetic resonance cholangiopancreatography; MRI: magnetic resonance imaging.

Sequences	Axial T2 TSE	Coronal T2 TSE	Coronal 3D MRCP
Plane	Axial	Coronal	Coronal
Pulse sequence	TSE	TSE	TSE
TR (ms)	440-450	540-550	1452
TE (ms)	80	80	650
Matrix	176 x 133	196 x 183	256 x 236
Slice thickness (mm)	2	6	2
Slice gap (mm)	1	0	-1
NSA	1	1	1
Flip angle	90°	90°	90°
Scan time (min)	1.30	1.15	4.24
SNR (%)	1	1	1

Statistical analysis was focused on the CNR and SNR for the stomach and duodenum as the primary outcome variables, with the type of oral contrast agent (pineapple juice, hematinic syrup, and date syrup) serving as the key discriminating variable. Mean and standard deviation were utilized for the descriptive analysis of the quantitative data, while frequency and proportion were applied to the categorical data. The analysis of variance (ANOVA) test was employed to assess the differences in the mean values of normally distributed quantitative variables across study groups, applicable when comparing more than two groups. For quantitative variables that did not follow a normal distribution, the Kruskal-Wallis test was used to compare medians and interquartile ranges (IQR) among more than two groups. Categorical variables across the study groups were compared using the Chi-square test. A p-value of less than 0.05 was deemed to indicate statistical significance. The analysis of data was conducted using the coGuide Statistics Software, Version 1.0 (Released 2020; BDSS Corp., India).

## Results

**Figure 3:**
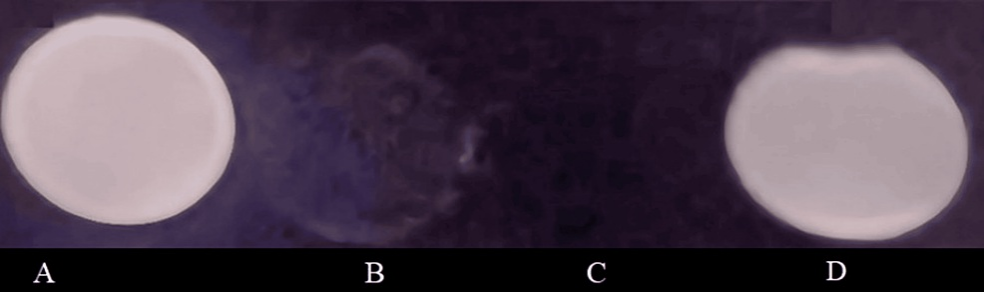
Assessment of phantoms infused with different oral contrast agents using T2-weighted MRI sequences Various contrast oral agents such as the following: (A) water, (B) oral hematinic syrup, (C) date syrup, and (D) pineapple juice.

In Figure 3, the in vitro evaluation revealed that date syrup and hematinic syrup exhibited hypointensity in T2W sequences. This indicates their potential as negative contrast agents for suppressing undesired signals from the gastrointestinal tract during MRCP.

 

**Table 2:** Comparative analysis of the demographic parameters and type of syrup in pineapple juice, iron syrup, and date syrup *, one-way analysis of variance (ANOVA); †, Chi-square test.

Demographic parameters	Type of syrup			p-values
	Pineapple juice (N = 30)	Hematinic syrup (N = 30)	Date syrup (N = 30)	
	Mean ± SD	Mean ± SD	Mean ± SD	
Age (years)	31.77 ± 8.98	30.43 ± 8.81	30.63 ± 8.64	0.8193*
Gender				
Male	21 (70.00%)	22 (73.33%)	22 (73.33%)	0.9461†
Female	9 (30.00%)	8 (26.67%)	8 (26.67%)	

Pineapple juice shows favorable demographic parameters compared to hematinic and date syrups. Specifically, pineapple juice exhibits higher mean age values (31.77 years) compared to hematinic (30.43 years) and date (30.63 years) syrups. However, the difference in mean age between the syrups is not statistically significant (p = 0.8193). Additionally, there is no significant difference in gender distribution among the three contrast agents (p > 0.05). Therefore, while pineapple juice may demonstrate slightly higher mean age values, the statistical analysis does not support the claim that it is definitively "better" than the other syrups in terms of demographic parameters alone.

**Figure 4:**
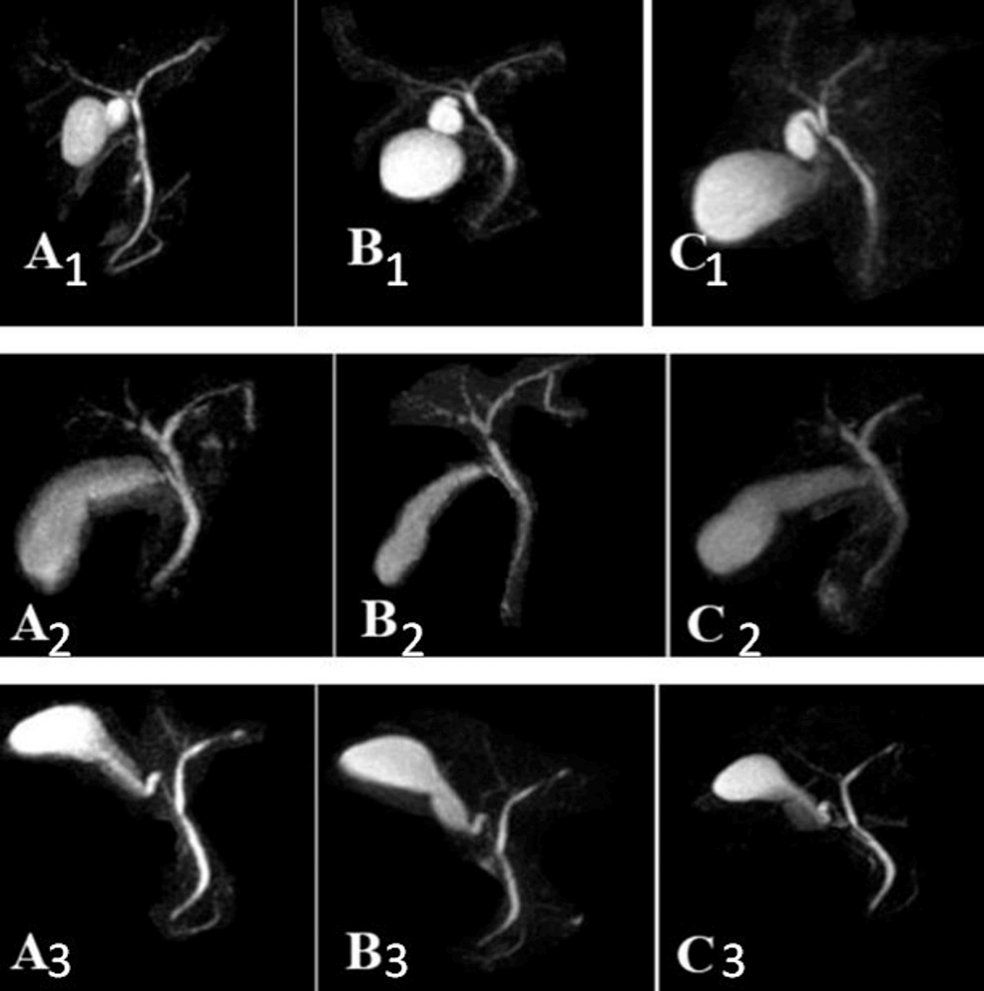
T2-weighted MRCP imaging was performed on various patients who were administered different oral agents (A) pineapple juice, (B) hematinic syrup, and (C) date syrup; all were administered at identical time intervals Three sample images from each group are presented: In column A, they are denoted as A1, A2, and A3, representing the pineapple juice group. In column B, they are labeled as B1, B2, and B3, corresponding to the hematinic syrup group. In column C, they are designated as C1, C2, and C3, indicating the date syrup group. In T2-weighted MRCP imaging using oral agents, Group A, represented by pineapple juice, exhibits a higher contrast-to-noise ratio compared to Groups B and C, represented by iron syrup and date syrup, respectively. This heightened ratio in Group A enhances the clarity and distinction of anatomical structures, potentially leading to more accurate imaging and diagnoses. Therefore, pineapple juice emerges as the preferred oral agent for T2-weighted MRCP imaging, offering enhanced contrast resolution and potentially superior diagnostic capabilities.

Figure 4 illustrates the outcomes of the in vivo assessment, where the efficacy of the three oral contrast agents was compared. Pineapple juice consistently exhibited higher SNRs in ROIs 4 and 5 in comparison to date syrup and hematinic syrup detailed in Table 3. Moreover, the CNR of pineapple juice surpassed those of the other two agents, suggesting superior contrast enhancement between the targeted structures and surrounding tissues.

 Our observations are listed in Table 3.

**Table 3:** Comparative analysis of the MRCP image parameters among different oral contrast agents *, One-way analysis of variance (ANOVA); †, Chi-square test The study uses multiple region of interest (ROIs) to assess different aspects of the images; ROI-1: This could be placed in the region of the common bile duct (CBD). ROI-2: Similar to ROI-1, ROI-2 could also be placed within the biliary tree, possibly in a different segment or branch from ROI-1. ROI-3: Positioned in the pancreatic duct. ROI-4 could be placed in a background region, such as an area outside of the body or within a region unaffected by the MRCP sequence; it serves as a reference for background noise or baseline signal intensity. ROI-5: Similar to ROI-4, ROI-5 might also be placed in a background region for reference; it could be positioned in a different area from ROI-4, providing additional background noise or signal intensity measurements.

Parameter	Type of syrup (mean ± SD)			p-value
	Pineapple juice (N = 30)	Hematinic syrup (N = 30)	Date syrup (N = 30)	
	Median (IQR)	Median (IQR)	Median (IQR)	
ROI-1	2897.12 (2248.98 to 3426.48)	2365.11 (2181.53 to 2981.24)	2365.11 (2181.53 to 2981.24)	0.2275 *
ROI-2	2489.23 (2060.43 to 3215.22)	2495.73 (2211.71 to 3212.84)	2489.23 (2204.98 to 3142.33)	0.9282*
ROI-3	2074.41 (1583.91 to 2227.08)	2175.50 (1651.18 to 2319.37)	2230.60 (1696.33 to 2448.39)	0.2688*
ROI-4	268.31 (209.15 to 298.39)	193.50 (119.3 to 236.54)	198.47 (150.4 to 277.0)	0.0052*
ROI-5	245.48 (203.38 to 256.26)	102.47 (97.48 to 119.28)	101.02 (97.23 to 119.28)	<0.001*
Mean CNR (ROI 1, 2, & 3)	6986.53 ± 19677.02	5009.1 ± 13637.16	4855.06 ± 13634.75	0.846†
SD CNR (ROI 1, 2, & 3)	8389.98 ± 33779.57	5209.34 ± 23231.5	4874.55 ± 23225.74	0.859†
Mean SNR ROI (4 & 5)	250.93 ± 62.85	155.5 ± 45.36	159.6 ± 46.45	<0.001†
SD SNR ROI (4 & 5)	34.76 ± 35.2	73.15 ± 48.04	73.01 ± 49.26	0.001†

ROI measurements (ROI-1 to ROI-5)

No significant differences were found in the medians between the syrups except for ROI-4 and ROI-5, where pineapple juice shows higher values (p < 0.01). Mean CNR (ROI 1, 2, & 3) and SD CNR (ROI 1, 2, & 3): No significant differences were found in the means or standard deviations between the syrups. Mean SNR ROI (4 & 5) and SD SNR ROI (4 & 5): Pineapple juice has the highest mean SNR, while hematinic and date syrups have lower means. Pineapple juice also has the lowest standard deviation, indicating less variability (p < 0.001). SNR stomach duodenum: Pineapple juice has the highest SNR, followed by iron and date syrups (p < 0.001). CNR CBD: Pineapple juice has the highest CNR, followed by date and iron syrups (p < 0.001).

## Discussion

MRCP, when assessing the pancreaticobiliary tree, is the preferred imaging modality. Bile and pancreatic juices are natural contrast agents because they involve the use of heavy T2W sequences that show stationary or slowly moving fluids as high signal intensity [11]. It is noninvasive, has high sensitivity and specificity for a range of pathologic conditions, does not require the administration of contrast agents, and has the potential for accurate visualization of the anatomic features of the pancreaticobiliary tree [11, 12]. A major drawback of the MRCP is the high-intensity signal due to the presence of stationary fluids in the abdominal cavity (stomach, duodenum, lymphatic, and renal collecting systems) [13]. Proper slice selection can eliminate the high signal intensity of the kidneys and lymphatics, but it is not possible to eliminate the high signal intensity of the GI tract because it is in the same plane as the pancreaticobiliary tree signal. Differentiating the anatomic features of the pancreaticobiliary tree can be challenging due to significant signal overlap caused by fluid in the stomach and duodenum. The use of a negative oral contrast medium can suppress undesired signals and enhance image quality [13].

Numerous naturally occurring compounds as well as commercial preparations of negative oral contrast agents have been tested. Although commercial preparations are costly, taste bad, and have adverse effects, they offer superior signal suppression. There have been mixed results when experimenting with pineapple juice, date syrup, and hematinic syrup. As a quick, accurate, and noninvasive substitute for diagnostic ERCP, MRCP has grown in popularity. Breathing artifacts, the superimposition of signals from the gastrointestinal tract, and low spatial resolution are additional limitations of MRCP. The restrictions have been greatly lessened by more recent fast imaging sequences with shorter acquisition times, thinner sections, respiratory gating, and breath-hold methods.

Frisch et al. [10], in their systematic review, recommended the use of oral solutions such as pineapple juice, ferumoxsil, or ferric ammonium citrate for suppression of GI signal in MRCP. Many other studies determined pineapple juice as a convenient oral contrast agent for MRCP [5, 9] [14-15]. With the use of lengthy TE sequences, the Mn concentration in all of the commercial juices was high enough to suppress the GI fluid signal [8].

We assessed the use of pineapple juice, hematinic syrup, and date syrup as potential negative oral contrast agents in our investigation. One rich source of iron is date syrup. The ferric (Fe3+) form of iron was present in date syrup. Due to its extremely high molar susceptibility, which is comparable to that of manganese, the ferric form of iron exhibits paramagnetic characteristics.

It was discovered that just 1.9 mg/dl of manganese is needed for effective gastrointestinal tract signal reduction. Hiraishi et al. [6], in "Blueberry juice: preliminary evaluation as an oral contrast agent in gastrointestinal MR imaging," suggested that the combined paramagnetic ion concentration of 2.9 mg/dl (iron plus manganese concentration) in date syrup is adequate for effective gastrointestinal tract signal suppression because the molar susceptibilities of iron and manganese are similar. Date syrup demonstrated exceptional T2-shortening effects and a very low SNR on T2W and single-shot MRCP images, according to our in vitro study. Govindarajan et al. [11] concluded that date syrup can be used as a negative oral contrast agent for GI signal suppression during MRCP to improve visualization of various pancreaticobiliary structures. Ferric ammonium chloride (hematinic) and ferric ammonium citrate were also found to be a good MR contrast agent [12, 16, 17].

We gave only oral negative contrast agents to the patients to suppress fluid in the stomach because it is required only to nullify the GI tract signal and not to fully distend in their stomach. We waited for the oral negative contrast agent to coat the stomach and duodenum, thereby reducing signals from the GI tract. It took at least half an hour after the patient consumed the contrast agent due to its viscous nature. Furthermore, none of the patients had reflux into the papilla due to the viscous nature of the syrup. As far as we are concerned, there haven't been any documented instances of date syrup toxicity or adverse effects in the literature. This study confirmed the absence of side effects. The amount of pineapple juice, date syrup, and hematinic syrup administered is less than the recommended daily intake amount.

The study investigating the effectiveness of pineapple juice, date syrup, and hematinic syrup as oral contrast agents for enhancing MRCP image quality provides valuable insights but also presents several limitations that should be acknowledged. Conducting the study at a single medical center might introduce biases related to patient selection and institutional practices, highlighting the need for multicenter studies with more diverse populations. Moreover, the exclusion criteria used in the study, which excluded individuals with specific medical conditions and contraindications to MRI, may restrict the relevance of the findings to real-world clinical situations. Additionally, the lack of a control or comparator group poses challenges in evaluating the comparative effectiveness of the oral contrast agents against standard imaging protocols or alternative contrast agents. Moreover, the subjective nature of image analysis by radiologists introduces variability and subjectivity into the assessment of MRCP image quality and contrast enhancement. Long-term follow-up and evaluation of clinical outcomes were also lacking, limiting the understanding of the sustained impact of the oral contrast agents on patient management.

## Conclusions

This study showed that 100 mL of date syrup and 30 ml of hematinic syrup diluted to 200 ml with water and 200 mL of pineapple juice when used as a negative oral contrast agent for MRCP suppresses undesired signals from the gastrointestinal tract and improves visualization of the common bile duct, cystic duct, and pancreatic duct. This effect is due to the high paramagnetic ion concentration in pineapple juice, which has excellent T2-shortening effects. In a few volunteers, the caliber of the pancreatic duct increased after the consumption of pineapple juice. We propose that this effect is probably due to the acidic nature of the juice. We found in our in vivo study that pineapple juice was still the best currently available contrast agent. We propose that date syrup as well as hematinic syrup, which are a rich source of iron, can be used as a suitable negative oral contrast agent in MRCP. This is the only study comparing the image quality of various oral negative contrast agents and also the image quality in visualizing the pancreaticobiliary tree in T2W and MRCP sequences which were fulfilled, and pineapple juice was found to have the lowest SNR and highest CNR giving the best image contrast and quality. Further studies need to be undertaken with qualitative and quantitative analysis of pineapple juice to find the nature and concentration of iron in it. Further large-scale vivo evaluation of pineapple juice in improving the quality of MRCP images needs to be done to translate these findings of research into clinical benefit.
